# Epidemiological patterns of traumatic musculoskeletal injuries and non-traumatic disorders in Japan Self-Defense Forces

**DOI:** 10.1186/s40621-018-0150-2

**Published:** 2018-05-01

**Authors:** Masatoshi Amako, Yoshiyuki Yato, Yasuo Yoshihara, Hiroshi Arino, Hiroshi Sasao, Osamu Nemoto, Tomohito Imai, Atsushi Sugihara, Satoshi Tsukazaki, Yutaka Sakurai, Koichi Nemoto

**Affiliations:** 1 Department of Orthopaedic Surgery, National Defense Medical College, 3-2 Namiki, Tokorozawa, Saitama 359-8513 Japan; 2 Department of Orthopaedic Surgery, Japan Self-Defense Forces Central Hospital, Setagaya, Tokyo, Japan; 3 Department of Orthopaedic Surgery, Japan Self-Defense Forces Sapporo Hospital, Sapporo, Hokkaido Japan; 4 Department of Orthopaedic Surgery, Japan Self-Defense Forces Hospital Kure, Kure, Hiroshima Japan; 5 Department of Orthopaedic Surgery, Japan Self-Defense Forces Hospital Yokosuka, Yokosuka, Kanagawa Japan; 6 Department of Public Health and Preventive Medicine, National Defense Medical College, Tokorozawa, Saitama Japan

**Keywords:** Musculoskeletal injury, Non-traumatic musculoskeletal disorder, Epidemiological surveillance, Fracture risk, Body mass index

## Abstract

**Background:**

The epidemiological patterns of musculoskeletal injuries or disorders in military personnel have not been well documented and a better understanding is required for proper preventative measures and treatment. Here, we investigated musculoskeletal injuries or disorders among members of the Japan Self-Defense Forces.

**Methods:**

All orthopedic patients (*n* = 22,340) who consulted to Japan Self-Defense Forces Hospitals were investigated for their type of injury or disorder, the injured body part, the mechanism, and the cause of injuries.

**Results:**

Thirty-nine percent of the cases were classified as traumatic injuries, and 61% were classified as non-traumatic disorders. Of the traumatic injury patients, the injured body part was the upper extremity in 32%, the trunk in 23%, and the lower extremities in 45% of the cases. The most common injured body location was the knee followed by the hand/finger and ankle. Exercise was the most common cause of injury, followed by traffic accident and military training.

Contusions were the most common traumatic injuries, followed by sprains and fractures. Of non-traumatic disorders, the lower extremities were reported as the injured part in 43% of the disorders. Lumbar spine disorders were the most common non-traumatic disorders, followed by tendon and joint disorders.

**Conclusions:**

Over one-third of orthopedic cases among members of the Japan Self-Defense Forces are traumatic injuries, with the knee being the body part most commonly injured and exercise being the leading cause of injury.

## Background

Current recommendations suggest that engaging in regular physical activity reduces cardiovascular events and many chronic diseases (Haskell et al., 2007). Increasing the amount of physical activity also increases the risk of musculoskeletal injuries or disorders. The Japan Health Labour and Welfare Ministry reported that fracture and joint disorder were major reasons for the need of long-term nursing care in Japan (Japan Health, Labor and Welfare Ministry, 2017). However, the prevalence musculoskeletal injuries or disorders is unclear. Recently, epidemiological studies of musculoskeletal disorders were conducted in Japan (Muraki et al., 2010; Yoshimura et al., 2009; Yoshimura et al., 2011; Tsunoda et al., 2013), with elderly subjects as the main population of their studies and degenerative disorders, such as osteoarthritis, spondylosis, and osteoporosis, examined. Musculoskeletal injuries or disorders are a common health problem for young populations, and a preventive system should be required using epidemiological data. Sports injury surveillance, as an epidemiological study, has been conducted in young athletes (Fukubayashi et al., 2010; Fukubayashi et al., 2012; Fukubayashi et al., 2013). However, the military population may also be ideal for obtaining epidemiological data of musculoskeletal problems. The United States Military has performed large injury surveillance studies, and the biggest health problem was traumatic injuries, especially lower extremity, training-related injury (Jones et al., 2010; Jones et al., 1993). Military medical corps in other foreign nations have also reported injury surveillance data (Frilander et al., 2012; Taanila et al., 2009). However, no previous studies of musculoskeletal disorders in a young military population in Japan. The purpose of the present study was to survey musculoskeletal injuries and disorders among military personnel in the Japan Self-Defense Forces, to analyze risk factors, and to compare to other athletic, military, and Japanese populations. Moreover, we also identified risk factors of fracture in the traumatic injury, and we hypotheses the risk factors of fracture were obesity and/or smoker. Ultimate aim of this study was to minimize these factors to prevent such musculoskeletal injuries or disorders in the Japanese Self Defense Forces members.

## Methods

With approval of our institutional review board (IRB No. 895), a longitudinal cohort study of musculoskeletal injuries and disorders was investigated in 13 Japan Self-Defense Forces Hospitals between 2008 and 2012. All patients with musculoskeletal problems were referred to the orthopedic outpatient clinic in the hospitals. Review sheets were provided at the first visit, and patients were asked to complete the review sheets on a voluntary basis and provide written informed consent. The review sheet included 17 questions concerning a time and date, age, sex, height, weight, rank, branch, uniformed service, service years, right/left, cause and sites of disorders, mechanism of injury, past history, smoking, and alcohol habits. These questions included several options, and patients chose the options before visiting to surgeons. The options of “cause of injury” included exercise, military training, repairing work, traffic accident, activity of daily living, and others. Exercise included sports in a club team, personal fitness, and jogging. On the other hand military training included official training during wearing boots, helmet, and uniform. The options of “mechanism of injury” included sprain, bruising, falling, outstretched, squeezing, lifting objects, cuts, being stepped on and others. After a medical examination, orthopedic surgeons reported the diagnosis on the review sheets. The data was collected and analyzed at our institution.

The inclusion criteria for this study were patients with musculoskeletal problems who were associated with the Japan Self-Defense Forces, including cadets. The exclusion criteria were retired members, civilian members, service members’ families, unconscious patients, patients without written informed consent, returning patients with the same diagnosis over a couple of months, patients with injuries to the head or face, and patients without musculoskeletal injuries or disorder.

We divided the patients with musculoskeletal problems into two groups: the traumatic injury group and the non-traumatic injury group. The traumatic injury group is defined as patients with bodily harm resulting from acute exposure to external forces, including fracture, joint dislocation, sprain, and contusion. The non-traumatic musculoskeletal disorder group is defined as patients with injury-related musculoskeletal conditions resulting from the cumulative effects of smaller amplitude forces that occur with over-training, repetitive movements, and forceful actions, including osteoarthritis, tendinitis, non-traumatic lower back pain, fatigue fracture, and entrapment neuropathy.

We focused on fracture in this traumatic injury group, and divided the patients into two additional groups: the fracture group and the non-fracture group. Risk factors in regards to gender, body mass index (BMI), age, type of uniformed service, rank, smoking, and alcohol habit were evaluated. BMI was divided into 4 groups based on the World Health Organization (WHO, BMI classification 1994)(The World Health Organization BMI classification, 1994): underweight (BMI < 18.5), normal weight (18.5 ≤ BMI < 25), overweight (25 ≤ BMI < 30), and obese (30 ≤ BMI). The associations of the 4 different weight classes and fractures were assessed by multiple logistic regression models to estimate odds ratios (OR) and 95% confidence intervals (CI). All models were adjusted for age, type of uniformed service, rank, smoking, and alcohol habit, and physical grade.

*S*tatistical analyses were performed using Student’s t test, repeated measure ANOVA test, and the chi-squared test. A multivariable analysis was performed using logistic regression analysis, and the odds ratio (OR) and confidence intervals (CI) were obtained. We carried out the analyses using JMP 10 (SAS Institute Japan, Tokyo).

## Results

The total number of patients with musculoskeletal problems was 22,340 during the study period, including 1458 females, 20,718 males, and 164 non-responders. The mean age was 35.2 ± 10.8 years (range, 15–60 years). The mean weight was 69.2 ± 10.7 kg (range, 32–138 kg), the mean height was 170.2 ± 6.4 cm (range, 147–199 cm), and the mean body mass index was 23.8 ± 3.1 (range, 11.7–44.1). Of the patients, 14,432 were in the Grand Force, 5787 in the Maritime Force, 1285 in the Air Force, and 836 were cadets. The population by rank included 4105 officers, 12,292 enlisted, 4611 soldiers, and 836 cadets (non-responder 496). We obtained 26,837 musculoskeletal diagnoses during the study period, with 10,442 traumatic injuries (38.9%), and 16,395 non-traumatic disorders (61.0%) (Table 1).

**Table 1:** Comparison of baseline demographics of patients between traumatic musculoskeletal injury and non-traumatic disorder

		Traumatic injuries		Non-traumatic disorders	
Total No.		10,442		16,395	
Average age (y)		33.7	15–60 y	36.4	15-60y
Age distribution	15–19 y	573	5.5%	1093	6.7%
	20–29 y	3646	34.9%	4154	25.3%
	30–39 y	2864	27.4%	3789	23.1%
	40–49 y	2452	23.5%	4952	30.2%
	50–60 y	907	8.7%	2394	14.6%
	non-responder			13	0.0%
Sex	male	9701	93.2%	15,138	92.6%
	female	660	6.3%	1140	7.0%
	non-responder	81	0.8%	117	0.7%
Alcohol habit	drink everyday	2132	21.0%	3363	20.5%
	drink sometimes	6290	60.2%	9850	60.1%
	non-drinker	1472	14.5%	2314	14.1%
	non-responder	548	5.2%	868	5.3%
Smoking habit	smoker	3974	38.1%	6289	38.4%
	non-smoker	6138	58.8%	9564	58.3%
	non-responder	330	3.2%	542	3.3%
Uniformed service	Ground Force	6752	64.7%	11,066	67.5%
	Maritime Force	2593	24.8%	3988	24.3%
	Air Force	528	5.1%	967	5.9%
	non-responder	569	5.4%	374	2.3%
Rank	Officer	1603	15.4%	3418	20.8%
	Enlisted	5829	55.8%	8941	54.5%
	Soldier	2194	21.0%	3313	20.2%
	Cadet	572	5.5%	374	2.3%
	non-responder	244	2.3%	349	2.1%

The body location of the injury included 31.9% in the upper extremity, 23.1% in the trunk, and 45.0% in the lower extremities. The most common body location of injury was the knee followed by the hand/finger and the ankle. On the other hand, musculoskeletal disorders were reported 17.5% in the upper extremity, 40% in the trunk, and 42.5% in the lower extremity. The most common body location of the disorder was the lumbar spine, followed by the knee and the cervical spine (Table 2).

**Table 2:** Comparison of body location between traumatic injuries and non-traumatic disorders

		Traumatic injuries			Non-traumatic disorders			Total		
		*n*	%	%	*n*	%	%	*n*	%	%
Upper extremities	Shoulder	930	8.9	31.9	1194	7.3	17.5	2124	7.9	23.1
	Upper arm	95	0.9		488	3.0		583	2.2	
	Elbow	353	3.4		325	2.0		678	2.5	
	Forearm	96	0.9		54	0.3		150	0.6	
	Wrist	385	3.7		330	2.0		715	2.7	
	Hand/Finger	1473	14.1		475	2.9		1948	7.3	
Trunk	Cervical spine	583	5.6	23.1	1506	9.2	40.0	2089	7.8	33.5
	Thoracic spine	17	0.2		12	0.1		29	0.1	
	Thorax	933	8.9		405	2.5		1338	5.0	
	Lumbar spine	881	8.4		4642	28.3		5523	20.6	
Lower extremities	Hip	77	0.7	45.0	342	2.1	42.5	419	1.6	43.4
	Femur	361	3.5		587	3.6		948	3.5	
	Knee	1620	15.5		3263	19.9		4883	18.2	
	Lower leg	464	4.4		1189	7.3		1653	6.2	
	Ankle	1335	12.8		484	3.0		1819	6.8	
	Foot/Toe	839	8.0		1099	6.7		1938	7.2	
Total		10,442	100	100	16,395	100		26,837	100	100

Exercise was the most common cause of injury (48.8%), followed by traffic accident (14.8%) and military training (13.3%). Exercise was the most popular cause of injury in younger generations, and the occurrence rate was more than 50% in the second and the third decades. The rate of traffic accident rose as the generation rose, but those of military training were not difference among the generations. During activities of daily living (ADL) and repairing work, traumatic injury increased with age (Table 3).

**Table 3:** Cause of traumatic injury by decades of age

Age (y)	15–19		20–29		30–39		40–49		50–60		Total	
	*n*	%	*n*	%	*n*	%	*n*	%	*n*	%	*n*	%
Exercise	357	62.3	2111	57.9	1358	47.4	938	38.3	323	35.6	5087	48.8
Traffic accident	60	10.5	450	12.3	404	14.1	449	18.3	184	20.3	1547	14.8
Military training	72	12.6	535	14.7	388	13.6	295	12.0	94	10.4	1384	13.3
ADL	39	6.8	229	6.3	304	10.6	334	13.6	145	16.2	1051	10.1
Repairing work	29	5.1	245	6.7	269	9.4	273	11.1	101	11.2	917	8.8
Other	7	1.2	24	0.7	52	1.8	38	1.5	16	1.7	137	1.1
Non-responder	9	1.6	52	1.4	89	3.1	125	5.1	44	4.9	319	3.1

Injury classification revealed that contusion (29.7%) was the most common, followed by sprain (26.8%) and fracture (11.5%). These injuries showed similar occurrence across age decades, and, in particular, the ratio of fracture was around 12% of injuries in every decade of age. The prevalence of tendon and ligament injuries was higher in younger generations (Table 4). A comparison of occurrence by diagnoses in the non-traumatic disorders showed a higher rate with lumbar (27.7%), tendon (23.2%), and joint disorders (16.1%). Lumbar disorder was the most common in each generation, with the highest rate in the fourth decade. Joint disorder or periarthritis was the most common in the sixth decade, and the incidence was increasing with age. Ligament disorder, bone bruise or the fatigue fracture was the most common disorder in the second decade, and the incidence was decreasing with age (Table 5).

**Table 4:** Traumatic injury classification by decades of age

Age (y)	15–19		20–29		30–39		40–49		50–60		Total	
	*n*	%	*n*	%	*n*	%	*n*	%	*n*	%	*n*	%
Contusion	135	23.6	1054	28.9	844	29.5	695	28.3	270	29.8	2998	28.7
Sprain	160	27.9	964	26.4	778	27.2	669	27.3	230	25.4	2801	26.8
Fracture	67	11.7	413	11.3	316	11.0	301	12.3	109	12.0	1206	11.5
Ligament injury	94	16.4	497	13.6	315	11.0	212	8.6	47	5.2	1165	11.2
Tendon injury	51	8.9	310	8.5	299	10.4	266	10.8	114	12.6	1040	10.0
Joint injury	35	6.1	216	5.9	189	6.6	203	8.3	95	10.5	738	7.1
Dislocation	25	4.4	125	3.4	69	2.4	44	1.8	10	1.1	273	2.6
Periarthritis	2	0.3	28	0.8	31	1.1	35	1.4	23	2.5	119	1.1
Nerve injury	1	0.2	12	0.3	12	0.4	14	0.6	5	0.6	44	0.4
Incision wound	2	0.3	20	0.5	5	0.2	8	0.3	2	0.2	37	0.4
Other	1	0.2	7	0.2	6	0.2	5	0.2	2	0.2	21	0.2

**Table 5:** Non-traumatic musculoskeletal disorder classification by decades of age

Age (y)	15–19		20–29		30–39		40–49		50–60		Total	
	*n*	%	*n*	%	*n*	%	*n*	%	*n*	%	*n*	%
Lumbar disorder	256	23.4	1115	26.8	1225	32.3	1370	27.7	582	24.2	4548	27.7
Tendon disorder	276	25.3	1098	26.4	824	21.7	1079	21.8	529	22.0	3806	23.2
Joint disorder	140	12.8	566	13.6	511	13.5	915	18.5	501	20.8	2633	16.1
Cervical disorder	10	0.9	157	3.8	404	10.6	614	12.4	261	10.9	1446	8.8
Periarthritis	42	3.8	176	4.2	189	5.0	400	8.1	252	10.5	1059	6.5
Ligament disorder	129	11.8	442	10.6	191	5.0	180	3.6	69	2.9	1011	6.2
Bone bruise	121	11.1	216	5.2	104	2.7	70	1.4	40	1.7	551	3.4
Nerve disorder	23	2.1	102	2.5	121	3.2	127	2.6	67	2.8	440	2.7
Fatigue fracture	54	4.9	93	2.2	40	1.1	42	0.8	11	0.5	240	1.5
Tumor	15	1.4	49	1.2	72	1.9	58	1.2	41	1.7	235	1.4
Other	27	2.5	140	3.4	108	2.9	97	2.0	54	2.2	426	2.6
Total	1093	100.0	4154	100.0	3789	99.9	4952	100.0	2353	100.1	16,395	100.0

Traffic accident was the most common cause of fracture (19.2%). Military training was the most common cause of ligament injury (18.3%). Maintenance work was the most common cause of contusion (11.7%). Contusion, sprain, and fracture occurred the second most from traffic accident, while ligament, tendon, and joint injuries and dislocation occurred during military training.

The number of patients in the fracture group was 1206 (male, 1124; female, 78; non-responders, 4), while that in the non-fracture group was 9236 (male, 8612; female, 582; non-responders, 42). The weight group distributed as shown in Table 6. The body weight and the body mass index in the fracture group were significantly lower than that in the non-fracture group (*p* < 0.001). In regards to gender and the body weight class, males showed a significant difference between the groups (*p* < 0.001), but females did not. Uni-variable analysis revealed no difference in the smoking or alcohol habit between the two groups. Multivariable analysis revealed that BMI was the only significant risk factor (*p* < 0.001). A lower BMI correlated more strongly with fracture.

**Table 6:** Odds ratios for fracture according to sex and body weight class

			Underweight	Normal weight	Overweight	Obese
		*p* value	(*n* = 70)	(*n* = 6720)	(*n* = 2512)	(*n* = 434)
All injuries	(*n* = 10,442)	< 0.001	2.187 (1.390–3.329)	1.0 (reference)	0.917 (0.794–1.057)	0.584 (0.397–0.829)
Male only	(*n* = 9736)	< 0.001	2.977 (1.725–4.947)	1.0 (reference)	0.919 (0.793–1.061)	0.592 (0.403–0.842)
Female only	(*n* = 660)	0.7857	1.268 (0.504–2.774)	1.0 (reference)	1.087 (0.363–2.640)	N/A

## Discussion

An advantage of this study is that the sample was a young homogeneous population. Because military personnel are homogeneous and closed society, they are an appropriate population for epidemiological study, and many studies relating to musculoskeletal injury or disorder in this population have been published (Hauret et al., 2010). Since 1988, automated outpatient surveillance data has been available for the United States Military services, and that data is useful for determining the magnitude and causes of injury, identifying possible prevention targets, and monitoring trends among military personnel (Jones et al., 2010). In contrast, Japan Self-Defense Forces have not constructed such a surveillance system, and the incidence or outline of musculoskeletal problems is not clear. This study investigated musculoskeletal injuries or disorders in the large population of Japan Self-Defense Forces. This was the first epidemiological surveillance of young, athletic population of Japanese military members, This study revealed that body height and weight was very homogeneous, and all of the population belong to the Japan Self-Defense Forces. They were selected by entrance examination, and they undergo physical tests every year. The population could be representative of young and active adult individuals in Japan. However, these injuries are important in terms of loss of time in their duties or training.

The United States military reported that 74% of combat wounds occurred in the extremities during Operation Iraqi Freedom and Operation Enduring Freedom (Owens et al., 2007; Goodman et al., 2012; Belmont Jr et al., 2011). The leading causes of medical evacuation were musculoskeletal and connective tissue disorders (Cohen et al., 2010). United States Military surveillance showed that the most common location was the wrist, hand, and finger (13%), followed by the ankle (12%) (Jones et al., 2010). Non-combat injuries were reported by the United States Army Brigade Combat Team during Operation Iraqi Freedom, and the most common region of injury was the hand (17.5%), followed by the knee, lumbar spine, and ankle (13.5% each) (Belmont Jr. et al., 2010). Our study revealed that the knee was the most common injury location, followed by the hand/finger, and ankle. These slight variances can be explained by the character of the study population, but the common location of traumatic injury for young adults is the knee, hand/finger, and ankle. Approximately half of the injuries (48.8%) were sprain in the United States Military surveillance, followed by contusion (16.3%) and fracture (9.8%) (Belmont Jr et al., 2011). The reasons that sprain is so common in the United States Military are unclear, but the occurrence rate of fracture is around 10% in both the United States Military and Japan Self-Defense Forces.

Although musculoskeletal traumatic injury is a common problem, most studies have been conducted in rural villages in Japan, the target population of their studies was elderly people, and the degenerative disorders have been clarified in those studies (Muraki et al., 2010; Yoshimura et al., 2009; Yoshimura et al., 2011; Tsunoda et al., 2013). Few studies have examined the type of musculoskeletal injuries, body location, magnitude, and causes of the musculoskeletal problems for the younger population. The Japan Amateur Sports Association (JASA) also reported the sports related injury, and the most common sports related injury was fracture (27.9%), followed by sprain (24.5%) and contusion (24.5%) (Fukubayashi et al., 2010; Fukubayashi et al., 2012; Fukubayashi et al., 2013). However, our results demonstrated that the most common traumatic injury was contusion (28.7%), followed by sprain (26.8%) and fracture (11.5%). Since that population of JASA was composed of applicants to the sports insurance system, non-applicants were excluded in the report, and minor injuries were not included. Contusion or sprain was very common, but minor, and the occurrence of such minor injury has not been well documented.

Character of non-traumatic musculoskeletal disorders in young population has not been well-known. Nakamura reported a cross sectional study of chronic musculoskeletal pain in Japan, but their population was not so young. They focused on chronic pain, and chronic pain occurred most commonly in the lower back (Nakamura et al., 2011). Our study showed that the non-traumatic disorder was also localized mainly to the lumbar spine, followed by the knee, and the cervical spine. However, in younger generation the incidence of ligament disorder, bone bruise or fatigue fracture was relatively higher than other generation. Although acute trauma may be a factor in some cases, many musculoskeletal disorders result from the cumulative effects of smaller amplitude forces during exercise of military training. These forces occur with overtraining, overexertion, repetitive movement and activities, and forceful actions, and further study is required to identified risk factor of the disorders, and to develop preventive measures (Sasao et al., 2012),.

Our final goal of this study was to prevent musculoskeletal injuries or disorders in Japan Self Defense Forces. van Mechelen revealed the measures to prevent sports injuries form part of what is called the ‘sequence of prevention’ (van Mechelen et al., 1992). Firstly the extent of the sports injury problem must be identified and described. Secondly the factors and mechanisms have to be identified. The third step is to introduce measures that are likely to reduce the future risk and/or severity of sports injuries. Finally the effect of the measures must be evaluated by repeating the first step. This study was the first step to develop preventive measures, and we could make clear many epidemiological facts in our population; 45% of injury occurred in the lower extremities, especially in the knee, exercise was the most common cause of injury, contusion was the common injury classification, and lumbar spine was the most common body location of the disorders. We have to continue our study to identify the mechanism and risk factors, and introduce a preventive measure, and assess its effectiveness by repeating the first step.

Fracture occurred in 11–12% of the injured population in the present study, and the patients with fracture require a lot of time and expense to resume their duties (Matsuhashi et al., 2014). The increase in the prevalence of obesity may increase the fracture burden among young women and children, but not young men (Jordan et al., 2013; Lazcano-Ponce et al., 2009; Kessler et al., 2013; Rana et al., 2009). While active lifestyles have health benefits, our results highlight the importance of injury prevention practices in conjunction with physical activity recommendations, particularly among women. Our results indicate that the risk factor of fracture was low body weight among the male population. Among the high-activity population, a lower body weight may indicate lower bone density, and increased incidence of fracture during strenuous exertion of the upper and lower extremities.

A disadvantage of the study is that the population was composed of patients who visited the Self-Defense Forces Hospitals. Healthy people without musculoskeletal problems were not included in this study. The diagnoses were conducted at the initial visit, and may have changed during the treatment periods. All patients belong to Japan Self-Defense Forces, and the number of female patients was quite low (6.7%). This study did not investigate the magnitude of injuries or disorders.

## Conclusions

This study investigated musculoskeletal injuries or disorders in the large population of the Japan Self-Defense Forces. This was the first epidemiological surveillance of young, athletic population of Japanese military members, and character of musculoskeletal problems were obtained: 45% of injury occurred in the lower extremities, and the most common body location of injury was the knee, exercise was the most common cause of injury, contusion was the most common injury classification, the most common body location of the musculoskeletal disorder was the lumbar spine, and low BMI was only the risk factor for fracture in the traumatic injuries.

## Abbreviations

ADL: Activities of daily living
BMI: Body mass index
